# Effects of provision of water and nesting material on reproductive performance of native Moo Lath pigs in Lao PDR

**DOI:** 10.1007/s11250-018-1541-7

**Published:** 2018-03-13

**Authors:** Ammaly Phengvilaysouk, Jan Erik Lindberg, Viengsamai Sisongkham, Phonpaseuth Phengsavanh, Anna Jansson

**Affiliations:** 1 Livestock Research Center, National Agriculture and Forestry Research Institute, P.O. Box 7170, Vientiane, Lao People’s Democratic Republic; 2 Department of Animal Nutrition and Management, Swedish University of Agricultural Sciences, P.O. Box 7024, 750 07 Uppsala, Sweden; 3 District Agriculture and Forestry Office, Mai district, Phongsaly province Lao People’s Democratic Republic; 4 Department of Anatomy, Physiology and Biochemistry, Swedish University of Agricultural Sciences, P.O. Box 7011, 750 07 Uppsala, Sweden

**Keywords:** Growth, Fluid balance, Mortality, Weight, Welfare

## Abstract

This study investigated the effect of providing extra water and nesting material to Moo Lath sows on piglet survival and growth. Three treatments were evaluated in a randomized block design with six sows/treatment. In the Control treatment, sows were not provided with nesting material or extra water apart from that included in the feed (conventional smallholder practice). In treatment NM, nesting material was provided 1–2 days before expected farrowing. In treatment NMW, nesting material as in NM and extra water were provided ad libitum throughout the study. Data on sow feed and water intake, plasma protein concentration (TPP), body weight, and re-mating period, and on litter size, body weight, and survival of piglets, were collected for two reproduction cycles. NMW sows had higher water intake than Control and NM sows (14.7, 4.5, and 4.5 L/day, respectively, SE = 0.2). The weight loss from 2 weeks prior to farrowing until weaning was smaller in NMW than in NM and Control sows (16.0, 23.8, and 22.9 kg, respectively, SE = 0.9). TPP dropped from farrowing until 21 days of lactation in NMW sows, whereas it increased or was unchanged in NM and Control sows. The re-mating period was shorter and the number of litters/year was higher in NMW than in Control and NM sows (2.2, 2.0, and 2.0, respectively, SE = 0.01). Piglet mortality was lower in NMW than in Control and NM (9.5, 43.9, and 26.7%, respectively, SE = 4.9). Piglets in NMW were heavier at weaning and had higher daily weight gain than Control and NM piglets. It was concluded that providing water ad libitum and nesting material improved piglet survival and growth, and that providing water ad libitum improved sow physiological and reproductive fitness. However, provision of nesting material without access to ad libitum water might increase susceptibility to heat stress in sows.

## Introduction

Pig production in Lao PDR is primarily based on smallholder production systems, which supply about 85% of the total production (Lao Statistics Bureau 2013). A major problem on smallholder pig farms in Lao PDR is poor reproductive performance, i.e., low piglet growth rate (20–50 g/day) and high mortality (30–50%) (Phengsavanh et al. 2010; Chittavong et al. 2012a). Smallholder pig farms are predominantly extensive, low-input systems found mostly in rural regions (Phengsavanh et al. 2011). These systems use native breeds and take advantage of by-products and naturally occurring feed. Scavenging around the village, forests, and fallow fields is common (Phengsavanh and Stür 2006; Chittavong et al. 2012a), but some farmers keep animals in enclosures or pens (Chittavong et al. 2012a), especially during the cropping season (Phengsavanh et al. 2011).

Pigs are often fed the same diet, irrespective of stage of growth and reproduction, and have to compete for a limited amount of feed provided (Stür et al. 2008). Traditionally, farmers only provide water to pigs mixed with the feed (Chittavong et al. 2012b), a practice that may result in insufficient water intake and dehydration. Low water intake can decrease feed intake and milk production, and thereby piglet growth rate (Kruse et al. 2011). Moreover, provision of nesting material to permit nest building seems to be rare in smallholder systems (Wischner et al. 2009). Nesting can reduce stress in connection with farrowing and can have positive effects on stillbirth rate (Thodberg et al. 2002; Cutler et al. 2006).

The objective of this study was to investigate the influence of providing extra water and nesting material on piglet survival rate and growth performance in native Moo Lath pigs. The hypothesis tested was that free access to water and nesting material has positive effects on body weight and plasma volume of sows, stillbirth rate, piglet survival, and piglet growth performance.

## Materials and methods

### Location

An experiment was conducted at the Livestock Research Center (Nam Xuang), 44 km north of Vientiane City, Lao PDR, from July 2014 to December 2015. There are two seasons in this region, a dry season (November–April) and a rainy season (May–October), with a mean daily temperature of approximately 27 °C in both seasons (Lao Statistics Bureau 2013).

### Experimental design and treatments

Eighteen Moo Lath gilts were used in the experiment, which included three treatments. The gilts were arranged in a randomized complete block design, with three treatments and six replicates per treatment (Table 1). Gilts were blocked by expected time of farrowing, to minimize the effect of environmental conditions. Thus, gilts impregnated within the same month and by the same boar were allocated randomly to the three treatments within each of the three blocks.

**Table 1:** Treatments evaluated in a complete randomized block design using 18 sows (A1–A18) mated with two boars (F13 or F14). The treatments were no nesting material and no extra water (Control), nesting material provided but no extra water (NM), and both nesting material and extra water provided (NMW)

Block 1			Block 2			Block 3		
Control	NM	NMW	Control	NM	NMW	Control	NM	NMW
F13/A11	F13/A2	F13/A5	F13/A1	F13/A4	F13/A12	F13/A3	F13/A15	F13/A16
F14/A14	F14/A9	F14/A7	F14/A10	F14/A13	F14/A8	F14/A18	F14/A17	F14/A6

The treatments were (1) Control, where no nesting material and no extra water were provided; (2) NM, where nesting material was provided 1–2 days before expected farrowing, but no extra water was offered; and (3) NMW, where nesting material was provided 1–2 days before expected farrowing and water was provided ad libitum throughout the study. In treatment NMW, the extra drinking water was offered by a nipple connected to a graded bucket and water consumption was recorded daily. In both the NM and NMW treatments, 5 kg of rice straw per sow was provided as nesting material and sows were allowed to perform nest building by themselves. The nesting material was removed 3 days post farrowing.

In all treatments, farrowing supervision was provided, including cleaning the newborn piglets with a dry towel, disinfection of the navel (1 mL Dertodine, Pharmaceutical Factory No.3, Vientiane, Lao PDR), cutting of teeth, and iron injection 7 days post farrowing (1 mL iron dextran, General Drugs House Co. Ltd., Bangkok, Thailand). Moreover, each litter was provided with rice straw as bedding material (0.5 kg/piglet) in a secluded corner of the pen.

### Experimental animals and management prior to the study

Prior to the experiment, 20 Moo Lath gilts aged 6–8 months and with live weight (LW) 30–40 kg were purchased in the villages of Pak Ou and Sayabouly in northern Laos. All gilts were selected from six litters, to reduce genetic variability. Two Moo Lath boars from the same litter were purchased from a breeding station in Vientiane city. All animals were kept in individual pens (130 cm × 180 cm) in an outdoor open shelter with a roof. All gilts were offered the same diet. All pigs were vaccinated for classical swine fever (1 mL/pig, local vaccine produced by the Department of Livestock and Fishery, Lao PDR), de-wormed (1 mL/30 kg, Ivermec injection, General Drugs House Co. Ltd., Bangkok, Thailand) and given a vitamin A, D_3_, and E injection (1–2 mL/pig, Biotecnocem, Dallas, USA) before the start of the experiment.

The two boars were kept in pens near the gilts to stimulate estrus and all gilts were mated in their third estrus (LW 73 ± 23 kg). A maximum of three gilts were mated per boar per week, and a maximum of 10 gilts per boar. Eighteen pregnant gilts were retained for the experiment. The two gilts excluded were either not pregnant or too far out of synchronization with the other gilts. Gilts entered the study at 2 weeks prior to farrowing and the study was completed at weaning, at 45 days after farrowing.

### Feeding

A non-pelleted feed mixed with water was fed to sows in all treatments. The feed was composed of rice bran, maize, and soybean meal (Table 2) and offered at 3–4% of sow live weight, plus another 0.25 kg/piglet. Feed allowance was adjusted to maintain sows at a body condition score of 3 (which is considered optimal for breeding sows; Young and Aherne 2005). The feed was mixed with 4.5 L water/sow/day. From 2 weeks of age until weaning at 45 days, the piglets were provided ad libitum with a non-pelleted creep feed composed of maize and soybean meal (Table 2). A mineral and vitamin premix (mineral premix, Chanaphant Industry Co. Ltd., Bangkok, Thailand) was added to the diet (0.5% of total diet). Feed was provided twice daily (08:00 and 16:00 h).

**Table 2:** Ingredients and composition of diets fed to sows and piglets (from 2 weeks of age) subjected to conditions of no nesting material at farrowing and no extra water (Control), nesting material at farrowing but no extra water (NM), and nesting material at farrowing and water provided ad libitum during gestation and lactation (NMW)

	Sow diet	Piglet creep feed
Ingredients (%)		
Rice bran	50	–
Maize meal	32	80
Soy bean meal	18	20
Chemical composition		
Crude protein, %	14.8	16.4
Lysine, g/kg	8.1	8.3
Crude fiber, %	16.0	3.4
Calcium, %	0.1	0.2
Phosphorus, %	0.5	0.1
Digestible energy, MJ*	13.8	15.9

*Calculated energy value according to INRA (www.evapig.com, version 1.3.1.7)

### Sample collection and analyses

Data were collected from two reproduction cycles per sow. The number of days between matings was recorded. Number of piglets per litter (born, stillborn, dead within 3 days, and dead at weaning) was recorded. Individual weight of piglets was recorded at birth and at weaning after 45 days (mean piglet weight in each litter used for analysis). Weight of sows was recorded at mating, at 2 weeks before farrowing, and at weaning. Water consumption, feed offered, and refusals were recorded daily. Feed samples were collected at the beginning, middle, and end of the experiment (from farrowing until weaning). Feed samples were analyzed for dry matter (DM), ash, nitrogen (N), neutral-detergent fiber (NDF), and acid detergent fiber (ADF), according to AOAC (1990). Crude protein (CP) was calculated as N × 6.25.

Blood samples of about 5–7 mL were collected by venipuncture from the jugular vein into lithium-heparin tubes in the morning at 7 days pre-farrowing, on the first day of observed nest building, and at day 21 of lactation. The samples were refrigerated for 1 h and then centrifuged at 3500×*g* for 15 min. The plasma was collected and stored at − 20 °C until analysis of total plasma protein concentration (refractometer, Atago, Japan).

### Statistical analyses

Statistical analyses were performed using SAS version 9.4 (SAS Institute Inc. 2013). The General Linear Model (GLM) procedure was used to analyze the effect of treatments on post-farrowing reproductive performance, sow and piglet weight, number of piglets born, and piglet mortality. The model included:

$$ {Y}_{\mathrm{i}\mathrm{jk}}=\mu +{\alpha}_{\mathrm{i}}+{t}_{\mathrm{j}}+{e}_{\mathrm{i}\mathrm{jk}} $$

where *Y*_ijk_ = post-farrowing reproductive performance, sow and piglet weight, number of piglets born, and piglet mortality, *μ* = overall mean, *α*_i_ = effect of reproductive cycle, *t*_j_ = effect of treatment, *e*_ijk_ = random error. Total plasma protein concentration (TPP) was analyzed as repeated-measures data (Mixed procedure in SAS (2014)). The relationships between time points within sow were modeled using unstructured covariance. Due to the large variations sometimes observed in basal individual TPP levels, comparisons were only made *within* treatment. For these comparisons, the Tukey-Kramer test was used and the level of statistical significance was set to *P* < 0.05. Data presented are least squares (LS) mean ± standard error of the mean (SEM).

## Results

### Feed and water intake, body weight, and plasma protein concentration in sows

There was no difference in feed intake between the treatments, but sows provided with extra water and nesting material (NMW treatment) had significantly higher (*P <* 0.001) water intake than sows in the Control and NM treatments (Table 3). There were no differences between treatments in body weight from mating until weaning, but the weight loss from 2 weeks prior to farrowing until weaning was smallest in sows in treatment NMW (Table 3). In NMW sows, TPP decreased from farrowing until 21 days of lactation, whereas it increased or was unchanged in NM and Control sows (Table 4).

**Table 3:** Feed and water intake and body weight (BW) of sows from mating until weaning in the treatments with no nesting material at farrowing and no extra water (Control), nesting material at farrowing but no extra water (NM), and both nesting material at farrowing and water provided ad libitum during gestation and lactation (NMW)

	Treatment			SEM	*P* value
	Control	NM	NMW		
Feed intake, kg/day	3.3	3.2	3.3	0.1	ns
Feed intake, % of BW	3.6	3.7	3.7	0.1	ns
Water intake, L/day	4.5^b^	4.5^b^	14.7^a^	0.2	***
Weight at mating, kg	73.3	71.7	72.5	6.8	ns
Weight prior to farrowing^1^, kg	105.4	101.4	105.3	5.1	ns
Weight at weaning^2^, kg	82.5	77.7	89.4	4.9	ns
Body weight loss^3^, kg	22.9^a^	23.8^a^	16.0^b^	0.9	***

Different superscript letters within rows indicate significant difference at *P* < 0.05

*SEM* standard error of the mean, *ns* not significant

^1^At 2 weeks prior to farrowing

^2^After 45 days

^3^Difference between 2 weeks prior to farrowing and weaning

**Table 4:** Plasma protein concentration (g/L) in sows in the treatments with: no nesting material at farrowing and no extra water (Control), nesting material at farrowing but no extra water (NM), and both nesting material at farrowing and water provided ad libitum during gestation and lactation (NMW)

	Treatment		
	Control	NM	NMW
At 7 days pre-farrowing	73 ± 1	77 ± 1	83 ± 1
At first day of nest building	74 ± 2	82 ± 2^#^	77 ± 2*
At 21 days of lactation period	76 ± 2	83 ± 2^#^	76 ± 2*

*Significant difference from 7 days pre-farrowing (ANOVA, *P* = 0.006, Tukey *P* ≤ 0.1)

^#^Significant difference from 7 days pre-farrowing (ANOVA, *P* < 0.04, Tukey *P* = 0.16–0.40)

### Reproductive interval of sows and piglet performance

There was no difference in gestation period between treatments, but the re-mating period was shorter and the number of litters/year was higher in sows in treatment NMW than in sows in treatments Control and NM (Table 5).

**Table 5:** Reproductive interval in sows in the treatments with no nesting material at farrowing and no extra water (Control), nesting material at farrowing but no extra water (NM), and both nesting material at farrowing and water provided ad libitum during gestation and lactation (NMW)

	Treatment groups			SEM	*P* value
	Control	NM	NMW		
Gestation period, days	116	116	116	0.4	ns
Re-mating period, days	68^b^	66^b^	47^a^	1	***
Number of litters/year	2.0^b^	2.0^b^	2.2^a^	0.01	***

*SEM* standard error of the mean, *ns* not significant

Different superscript letters within rows indicate significant difference at *P* < 0.05

There was no difference in the number and proportion of born and stillborn piglets between treatments, but the mortality after 3 days was lower in NMW and NM than in Control*.* Moreover, at 45 days (weaning), mortality was lower in NMW than in both NM and Control (Table 6). The Control treatment had the highest mortality. In general, piglets that died both within 3 days and later had comparatively low birth weight, appeared less active, and could not compete successfully with the others when suckling. There was no difference in the weight of piglets at birth, but at weaning, piglets in treatment NMW were heavier and had higher average daily weight gain than piglets in NM and Control (Table 6).

**Table 6:** Performance of piglets from sows in the treatments with: no nesting material at farrowing and no extra water (Control), nesting material at farrowing but no extra water (NM), and both nesting material at farrowing and water provided ad libitum during gestation and lactation (NMW). *SEM* standard error of the mean

	Treatment			SEM	*P* value
	Control	NM	NMW		
Number of piglets/litter					
At birth	7.6	8.1	7.7	0.7	ns
Stillborn	0.5	0.6	0.4	0.2	ns
Born alive, but dead in 3 days	2.3^a^	0.7^b^	0.4^b^	0.2	***
Weaning	4.2^c^	5.4^b^	6.8^a^	0.3	***
Live weight					
At birth, kg	0.6	0.6	0.6	0.02	ns
At weaning (45 days), kg	6.0^b^	6.0^b^	7.0^a^	0.1	***
ADG^1^, g/d	118^b^	119^b^	143^a^	2	***
Mortality					
Stillborn, %	5.6	5.3	4.4	2.1	ns
At 3 days, %	31.7^a^	7.5^b^	5.2^b^	2.2	***
From birth to weaning, %	43.9^a^	26.7^b^	9.5^c^	4.9	***

Different superscript letters within rows indicate significant difference at *P* < 0.05

^1^Average daily gain

## Discussion

This study clearly demonstrates the benefits of ad libitum access to water in sows. Reproductive performance was markedly improved with ad libitum access to water, as re-mating period was shortened by 21 days, the number of piglets per litter at weaning was increased by more than 2.5-fold, and mortality at weaning was lowered to 9.5%, compared with 44% in the Control treatment. A 9.5% level of mortality is similar to that reported for European and North American production systems (Kirkden et al. 2013) and shows that it is possible, using fairly simple means, to improve performance in local smallholder systems. The provision of nesting material also improved the reproductive response, but the effect seemed to be restricted to increased survival of piglets during the first 3 days. Notably, provision of nesting material without access to water ad libitum induced loss of plasma volume (dehydration) in sows, which might make them more susceptible to, e.g., heat stress. Our hypothesis that free access to water and nesting material has positive effects on body weight and plasma volume of sows, stillbirth rate, piglet survival, and growth performance was therefore confirmed, but there were indications that provision of nesting material without water may not be optimal for sows.

In the treatment with ad libitum water provision, the sows drank almost 15 L/day, three times the allowance in the Control treatment. The level of intake corresponded to a water-to-feed ratio of 4.5 kg/day, which is of the same magnitude as observed in sows of breeds that are twice as large (5.8 kg/day; Kruse et al. 2011). Voluntary water intake is strongly affected by environmental factors, with ambient temperature and resulting evaporative losses being one such factor. Renaudeau et al. (2001) showed a doubling in water consumption when ambient temperature was increased from 20 to 29 °C, i.e., just above the temperature in the present study (27 °C). In the present study, the sows, especially those without extra water, were often observed lying down and with elevated breathing frequency, indicating that they were out of their thermoneutral zone. The importance of facilitating thermoregulation should not be underestimated. It has been shown that supplying chilled water (10–15 °C compared with 22 °C) can improve the performance of sows and their piglets (Jeon et al. 2006).

The loss of body weight from 2 weeks prior to farrowing to weaning was significantly lower (6 kg) in sows with ad libitum access to water than in sows with restricted water intake. Greater body weight loss in sows with restricted water intake has been reported previously (Leibbrandt et al. 2001). However, in the present study we also showed that sows supplied with water ad libitum were able to increase their plasma volume (indicated by lower TPP) during this period, in contrast to the sows in the other treatments. An increase in blood and plasma volume can be expected during gestation in normal, healthy sows (Matte et al. 1996). In contrast, in sows provided with nesting material (treatments NM and NMW), the plasma volume seemed to decrease during this period. This might be due to extra evaporative losses caused by the heat production from nesting activity and feeding if some straw was consumed, and the lack of possibility to restore these losses.

Ad libitum provision of water had marked positive effects on piglet survival and growth. Survival at weaning and weight gain was greatest in the treatment with water supplied ad libitum (NMW). The improved growth in NMW piglets was most likely a result of increased milk production in the sow, but part could be due to the piglets having the possibility to drink water. During the last 2 weeks before weaning, some piglets were observed drinking from the nipples. It has been shown that even very young piglets can drink up 200 mL/day (Fraser et al. 1988). Creep feed was available from 2 weeks and the possibility to drink may also have increased feed intake, but piglet feed intake was not measured. It is known that restricted water intake can affect voluntary feed intake (Leibbrandt et al. 2001).

Provision of nesting material increased the number of piglets that survived (i.e., did not die in) the first 3 days by 70% ((2.3–0.7)/2.3), but otherwise, there were no effects that could be linked to this treatment. In a study by Westin et al. (2014), weight at weaning was found to increase in systems providing 15–20 kg straw compared with 0.5–1.0 kg, but this effect could not be confirmed in the present study. There are conflicting results on the effect of nesting material and the risk of death in piglets (Kirkden et al. 2013). In one recent study comparing systems providing either 15–20 kg or 0.5–1.0 kg of straw, the number of piglets crushed was higher in the former system, but overall pre-weaning mortality of piglets born live was not affected by the treatments (Westin et al. 2015). However, piglet survival was improved in the present study, which could be due to nest-building behavior increasing oxytocin and prolactin levels, altering nursing behavior, and increasing the carefulness of sows when lying down (Yun et al. 2014).

The results clearly show that a system providing nesting material and water ad libitum should be recommended to farmers in Laos PDR. Rice straw is easy available during the wet season, cheap or sometimes even free. In the dry season, cattle farmers may have rice straw in storage. In remote villages there might be a lack of water, but in most cases, water can be collected from rivers, mountains, or even the ground. The main challenge now is to educate farmers on the importance of providing extra water.

In conclusion, the present study showed that providing water ad libitum and nesting material improved piglet survival and growth, and that providing water ad libitum also improved the physiological and reproductive fitness of the sow. However, provision of nesting material without ad libitum water access might increase the susceptibility to heat stress in sows. A management strategy including both nesting material and ad libitum water should therefore be recommended to farmers, both from a farm income perspective and an animal welfare perspective.
